# Diversifying *de novo* TIM barrels by hallucination

**DOI:** 10.1002/pro.5001

**Published:** 2024-05-09

**Authors:** Julian Beck, Sooruban Shanmugaratnam, Birte Höcker

**Affiliations:** ^1^ Department of Biochemistry University of Bayreuth Bayreuth Germany

**Keywords:** *de novo* protein design, hallucination, machine learning, small molecule binding site, (βα)_8_‐barrel

## Abstract

*De novo* protein design expands the protein universe by creating new sequences to accomplish tailor‐made enzymes in the future. A promising topology to implement diverse enzyme functions is the ubiquitous TIM‐barrel fold. Since the initial *de novo* design of an idealized four‐fold symmetric TIM barrel, the family of *de novo* TIM barrels is expanding rapidly. Despite this and in contrast to natural TIM barrels, these novel proteins lack cavities and structural elements essential for the incorporation of binding sites or enzymatic functions. In this work, we diversified a *de novo* TIM barrel by extending multiple βα‐loops using constrained hallucination. Experimentally tested designs were found to be soluble upon expression in *Escherichia coli* and well‐behaved. Biochemical characterization and crystal structures revealed successful extensions with defined α‐helical structures. These diversified *de novo* TIM barrels provide a framework to explore a broad spectrum of functions based on the potential of natural TIM barrels.

## 1 INTRODUCTION

Protein space is not limited to the sequences sampled by natural evolution but can be expanded through *de novo* protein design by creating new sequences (Huang, Boyken, & Baker, 2016). Basic principles to design idealized proteins from scratch have been defined, and a wide variety of *de novo* proteins with different topologies have already been generated (Dou et al., 2018; Doyle et al., 2015; Huang, Feldmeier, et al., 2016; Kim et al., 2023; Koga et al., 2012; Marcos et al., 2018; Minami et al., 2023; Pan & Kortemme, 2021; Yang et al., 2021). One important fold is the (βα)_8_‐ or triose‐phosphate isomerase (TIM) barrel, which is ubiquitous in nature and prominent in enzymes (Romero‐Romero, Kordes, et al., 2021; Sterner & Höcker, 2005). It is present in all classes of the Enzyme Commission except the translocase class. The structure is composed of eight alternating βα‐subunits, forming a central eight‐stranded, parallel β‐barrel encompassed by eight α‐helices (Wierenga, 2001). One of the key characteristics of this fold is the spatial separation of stability and catalytic function. Protein stability is achieved through the hydrophobic core of the barrel and the αβ‐loops situated at the N‐terminal ends of the β‐strands (Vijayabaskar & Vishveshwara, 2012). In contrast, the catalytically active residues are found at the C‐terminal ends of the β‐strands (Nagano et al., 2002). Typically, substrate binding occurs via a cavity formed at the central surface of the β‐sheet, which is supported by elongated βα‐loops on the top of the barrel (Thoma et al., 2000).

Since one prominent objective of *de novo* protein design is to create tailor‐made enzymes, the TIM‐barrel fold is an outstanding target. After decades of attempts to understand the principles of the TIM‐barrel fold, Huang, Feldmeier, et al. (2016) succeeded in building the first *de novo* TIM barrel from scratch, named sTIM11, thereby providing a TIM‐barrel scaffold that is free from any evolutionary biases paving the way for further investigations into the capabilities of this fold. In a highly rational fashion, the design problem was simplified by the introduction of a four‐fold symmetry and a restriction of the design approach based on geometrical constraints derived from the inner β‐sheet. Since then, the idealized sTIM11 with its minimal loops was subject to multiple modifications to increase folding, stability, and crystallizability, resulting in a *de novo* TIM‐barrel family with over 20 members (Kordes et al., 2022; Romero‐Romero, Costas, et al., 2021). Recently, the family of *de novo* TIM barrels was further expanded by a two‐fold symmetric design, leading to a distinctive curvature of the central β‐barrel and an overall ovoid shape of the barrel (Chu et al., 2022). Amidst the ongoing machine learning revolution and the emergence of AlphaFold2, numerous novel tools have been integrated into the realm of *de novo* protein design, diverging from traditional rational‐ and physics‐based approaches (Jumper et al., 2021). Nevertheless, the TIM‐barrel fold remains a promising design target, as new methodologies have already been utilized to expand the *de novo* TIM‐barrel family. Notably, Anand et al. (2022) harnessed a potential learned neural network, while Goverde et al. leveraged AlphaFold2 and proteinMPNN to successfully redesign sTIM11 (Dauparas et al., 2022; Goverde et al., 2023; Jumper et al., 2021). These efforts led to a significant expansion of the sequence space of *de novo* TIM barrels and a deviation from the so‐far established sequence symmetry.

In addition to redesign approaches, neural networks have shown their ability to generate entirely novel proteins from scratch. An approach called hallucination utilizes the structure prediction software RoseTTAFold for the optimization of random sequences that result in the generation of diverse proteins with a wide range of sequences and predicted structures (Anishchenko et al., 2021; Baek et al., 2021). Expanding on this, two additional approaches called constrained hallucination and inpainting utilize initial information such as functional sites to construct diverse protein frameworks without the need to predefine a fold or secondary structure (Wang et al., 2022). By fine‐tuning RoseTTAFold for denoising tasks, a new approach known as RFdiffusion was developed (Watson et al., 2023). This method can tackle multiple protein design tasks, including unconditional and topology‐constrained protein monomer design. To showcase the potential of RFdiffusion in generating targeted folds, the authors designed several TIM barrels. However, RFdiffusion only generates backbones, and its sequence design relies on proteinMPNN (Dauparas et al., 2022).

Despite the growing number of *de novo* TIM‐barrel structures with these new artificial intelligence (AI) tools, all generated *de novo* TIM barrels still lack the feature of cavities, pockets, or extended loops compared to natural TIM barrels, which exhibit a wide variety of structural elements in their βα‐loops. Thus, to create functionalized *de novo* TIM barrels, incorporating structural extensions or hydrophobic pockets becomes essential. Numerous attempts have been made to diversify the idealized structure of sTIM11. The already‐mentioned ovoid‐shaped barrel was designed with non‐structured loops capable of adopting diverse conformations (Chu et al., 2022). In a separate study, Wiese et al. (2021) introduced a small helix into the βα‐loops of the barrel. Building on this concept, Kordes et al. (2023) implemented a larger helix–loop–helix motif. In another work, Caldwell et al. (2020) split the TIM barrel and fused a designed ferredoxin fold, creating a homodimer with a cavity which was functionalized downstream with a metal binding site. All these endeavors demonstrate the versatility of *de novo* TIM barrels in accommodating different structural motifs while emphasizing the importance of diversifying their idealized structure.

In this work, we aimed to expand the *de novo* TIM‐barrel family by introducing secondary structural elements to enhance its surface area and create a cavity. Taking advantage of state‐of‐the‐art machine learning methods, we hallucinated extensions and optimized the sequences with proteinMPNN, whereby generating *de novo* TIM barrels with two or three helical extensions. These designs were analyzed through biophysical and structural characterization.

## 2 RESULTS

### 2.1 Constrained hallucination incorporates helical hairpins into sTIM11‐SB

**FIGURE 1:**
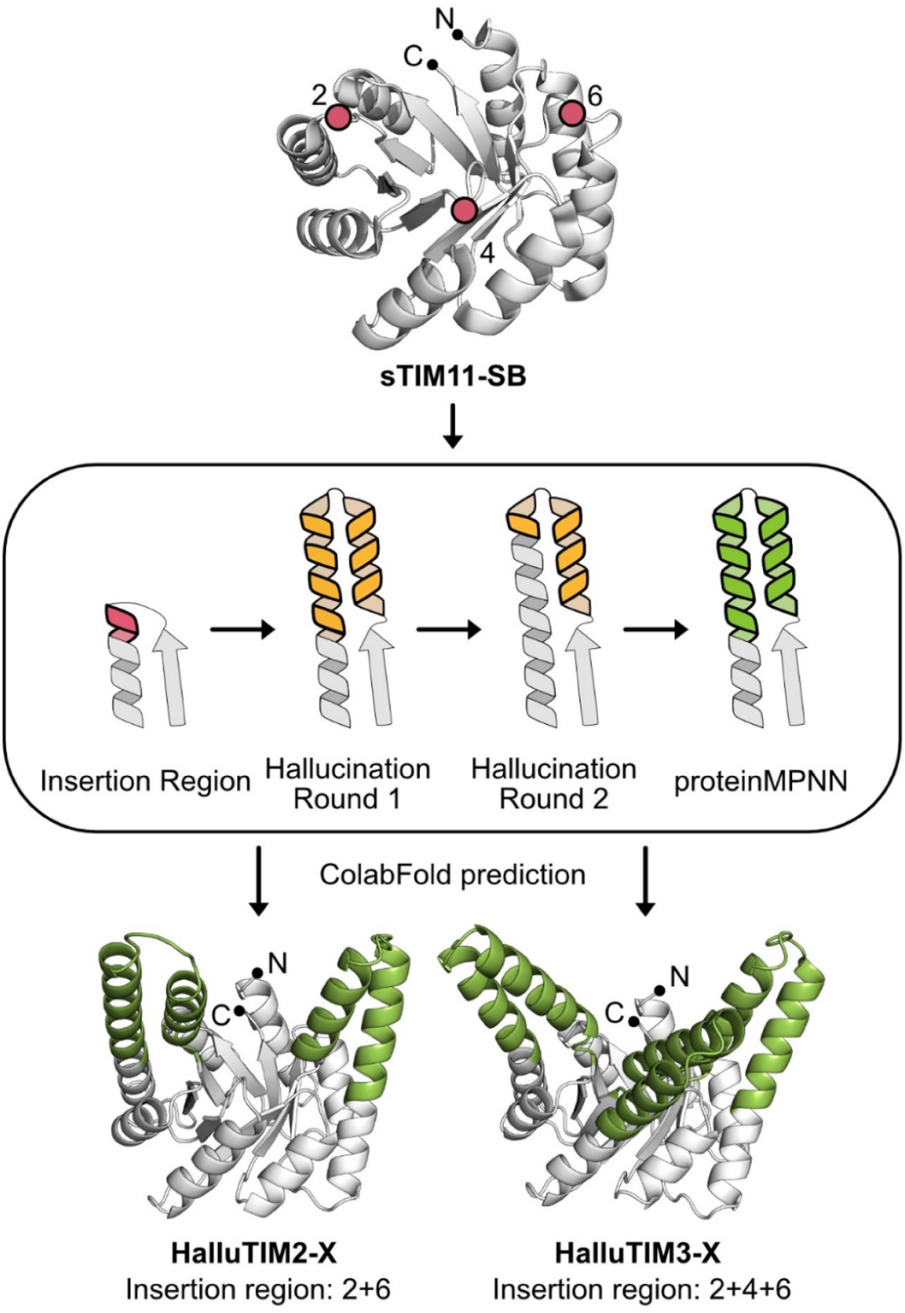
Design workflow for the extensions in sTIM11‐SB. For the hallucination of extensions sTIM11‐SB (PDB‐ID: 7OSU), displayed in white and as cartoon representation with black dots highlighting the termini, was used as a base. Three insertion sites were defined within the second, fourth, and sixth loop, marked with a red dot. For the constrained hallucination as shown in the central panel, the first turn of the outer α‐helix (in red with thick outline) and the loop to the inner β‐strand were used as the insertion region. During round one of constrained hallucination α‐helical extensions were obtained on top of the barrel (in yellow with thick outline). Within round two, the newly obtained outer α‐helix was kept fixed except for the last turn and only the smaller α‐helix was hallucinated again, highlighted with the thicker black outline. After this a sequence optimization of the entire hallucinated fragment was performed (in green with thick outline) and the structure of the designs were predicted with ColabFold. For constrained hallucination, either insertion region one and three or all were used, resulting in HalluTIM2‐X with two α‐helical extensions or HalluTIM3‐X with three extensions (in green).

For the diversification experiment, we used the *de novo* TIM barrel sTIM11‐SB as the base scaffold. This variant contains a stabilizing salt bridge cluster in the lower part of the β‐barrel (Kordes et al., 2022). As a method, we applied the constrained hallucination approach from Wang et al. (2022) and chose as insertion regions the three elongated βα‐loops on the C‐terminal side of the β‐strands (Figure 1). We decided to hallucinate either two extensions in the second and fourth quarter of the barrel or combine these with an additional one in the third quarter opposite to the termini to increase the chances of building up a cavity. The hallucinated fragments within these models turned out to be an elongation of the outer α‐helix by multiple turns as well as the generation of a small α‐helix above the inner β‐strand resulting, in a helix–loop–helix motif (Figure 1). Notably, this topology of the hallucination is present not only in the best but also in most of the designs. To estimate the backbone diversity of the designs, we calculated the TM‐score of each design against all others within the initial round of hallucination (Zhang & Skolnick, 2005). The lowest TM‐scores within each dataset were found to be approximately 0.81, indicating a low backbone diversity. The highest deviation in the generated structures is found within the region above the inner β‐strands. Here, not always a continuous α‐helix for the full helix–loop–helix motif is formed but sometimes only a loopy connection to the outer elongated helix. Interestingly, all these designs showed lower pLDDT‐ scores than the ones with a fully formed helix–loop–helix motif and were thus discarded during the filter process. To further increase the quality of our designs, we performed a second round of constrained hallucination with the top scoring designs. Hereby, the elongated outer α‐helix was kept fixed except for the last turn, and the design was focused on the smaller α‐helix packed against the elongated one (Figure 1). With this strategy, we were able to improve the average pLDDT of all modeled designs, but the top scoring designs showed only a slight improvement as the original input already had a tight packing of the α‐helices against each other. Since the second round of constrained hallucination did not significantly improve the best designs, we did not perform a third round but instead optimized the sequences of the extensions using proteinMPNN (Dauparas et al., 2022) (Figure 1). After the prediction of all generated sequences with ColabFold (Mirdita et al., 2022), six designs were selected for experimental characterization based on the average pLDDT score and the packing of the hallucinated α‐helices. We chose three designs for each insertion site combination (Tables S1 and S2). The constructs were named HalluTIMX‐X, whereby the first X corresponds to the number of extensions and the second X differentiates the constructs within the same category (Figure 1).

### 2.2 Experimentally tested HalluTIM variants show increased helicity and thermostability

**FIGURE 2:**
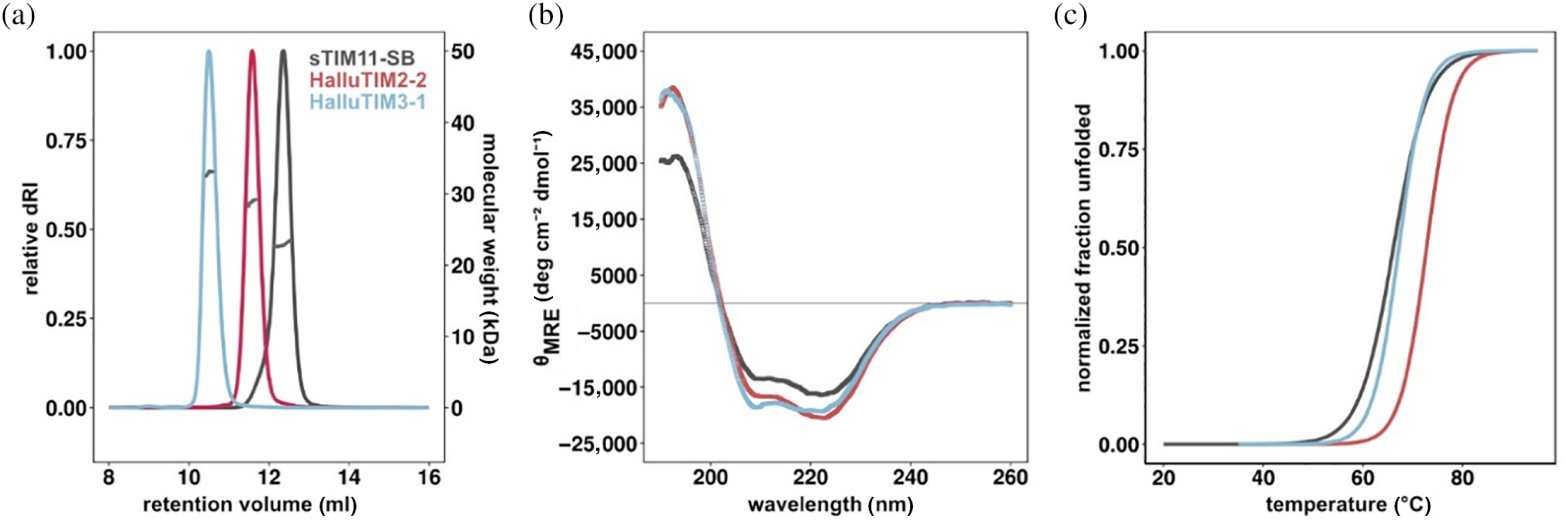
Biochemical analysis of HalluTIMs in comparison with the base scaffold. Experimental characterization of HalluTIM2‐2 (in red), HalluTIM3‐1 (in blue) and sTIM11‐SB (in gray). (a) Elution profile of size exclusion chromatography‐multi angle light scattering measurements showing the normalized relative differential refractive index as solid line and the calculated molar mass as data points in dark gray within the corresponding peak. With each extension, the hydrodynamic radius and molecular weight increases. For experimentally determined masses see Table S3. (b) Circular dichroism spectra show increases in α‐helicity for both HalluTIMs compared to the base scaffold. (c) Thermal unfolding followed by circular dichroism shows an increase in stability of the designs compared to sTIM11‐SB. For melting points and Δ*G*_25°C_ values, see Table S3. dRI, differential refractive index.

After heterologous expression in *Escherichia coli*, all designs were found in the soluble fraction of the cell extract and could be purified to homogeneity. All designs except HalluTIM2‐3 showed a homogenous peak corresponding to monomeric proteins and an increased hydrodynamic radius in comparison to the base construct sTIM11‐SB in size exclusion chromatography‐multi angle light scattering (SEC‐MALS) analysis (Figures 2a and S1). HalluTIM2‐3 displayed two species with slightly different hydrodynamic radii. Each experimentally determined molecular weight corresponds well to the theoretical monomeric molecular weight (Table S3). Analysis of the secondary structure content by circular dichroism (CD) spectroscopy revealed the spectra of well folded proteins. In comparison to the basic scaffold sTIM11‐SB all HalluTIMs, except HalluTIM2‐3 and HalluTIM3‐2, showed an increase in α‐helicity (Figures 2b and S1), which indicates proper formation of the hallucinated extensions. However, no major differences in the increase in α‐helicity between the constructs are observable, despite the introduction of a different number of helical extensions. To investigate if the hallucinated extensions influence protein stability, we followed the thermal unfolding by CD for all proteins (Figures 2c and S1). Interestingly, we observed a similar or even a higher melting temperature for all HalluTIMs, except HalluTIM2‐3, in comparison to the base scaffold (Table S3). By calculating unfolding parameters for each protein, we obtained similar or higher Δ*G*_25°C_ for all HalluTIMs except HalluTIM2‐3 in comparison to sTIM11‐SB (Table S3), indicating that the extensions stabilize the entire TIM‐barrel protein. Interestingly, these changes in Δ*G*_25°C_ are caused mainly by a change in cooperativity. In addition, we checked on the reversibility of unfolding by collecting CD spectra after the melting process (Figure S2) observing that all HalluTIMs maintained the reversibility of the base scaffold.

### 2.3 Crystal structures of two HalluTIMs validate the formation of novel extensions

**FIGURE 3:**
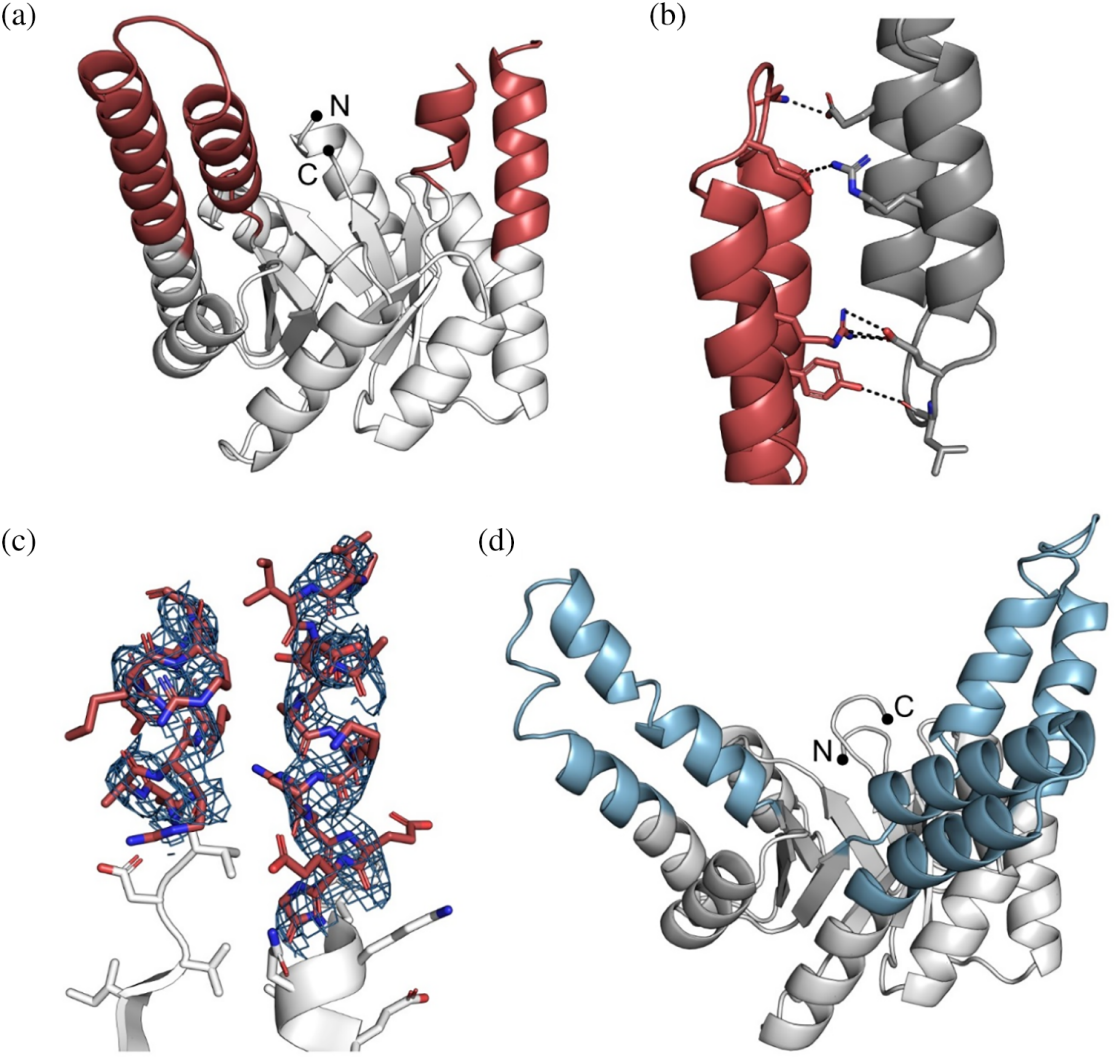
Structural details of HalluTIM2‐2 and HalluTIM3‐1. All structures are displayed in cartoon representation with black dots highlighting the termini. The base scaffold is shown in white. Extensions of HalluTIM2‐2 and HalluTIM3‐1 are colored in red and blue, respectively. (a) Overall structure of HalluTIM2‐2 (chain A, PDB‐ID: 8R8N). (b) Resolved helical extension of HalluTIM2‐2 forms multiple crystal contacts with its symmetry mate (in gray). Contacts such as polar interactions and hydrogen bonds are shown as black dashed lines. (c) Partially resolved second extension in HalluTIM2‐2 shown as stick representation in red with the corresponding electron density in blue (2Fo‐Fc map contoured at 1.0 RMSD). (d) Overall structure of HalluTIM3‐1 (PDB‐ID: 8R8O).

To gain more insights and validate the successful incorporation of the hallucinated extensions, we crystallized HalluTIM2‐2 (Protein Data Bank‐Identifier (PDB‐ID): 8R8N) and HalluTIM3‐1 (PDB‐ID: 8R8O). The cartoon representations are shown in Figure 3 and the crystallographic details are listed in Table S6. Within the crystal structure of HalluTIM2‐2, the α‐helical extension at position 1 is resolved entirely; it forms multiple crystal contacts with itself (Figure 3b). The second extension at position 3 is not involved in any crystal contacts, and one helical turn before and after the loop could not be resolved (Figure 3c). In the case of HalluTIM3‐1, the crystal structure shows all three intended hallucinated extensions in their entirety, verifying their successful incorporation into sTIM11‐SB. One minor deviation between HalluTIM3‐1 and the base scaffold is observed within the N‐terminal α‐helix of the barrel, as these residues do not form a continuous α‐helix. Notably, for both crystal structures, a significant number of crystal contacts are formed within the resolved α‐helices that had been optimized with proteinMPNN. In the case of HalluTIM3‐1, the crystal has an uncommonly high solvent content of 78% (Matthews coefficient: 5.6) (Figure S3), that may influence the quality of the data and in combination with a certain flexibility of the extensions, lead to the rather noisy diffraction data.

### 2.4 Solution states match structures despite crystal contacts

**FIGURE 4:**
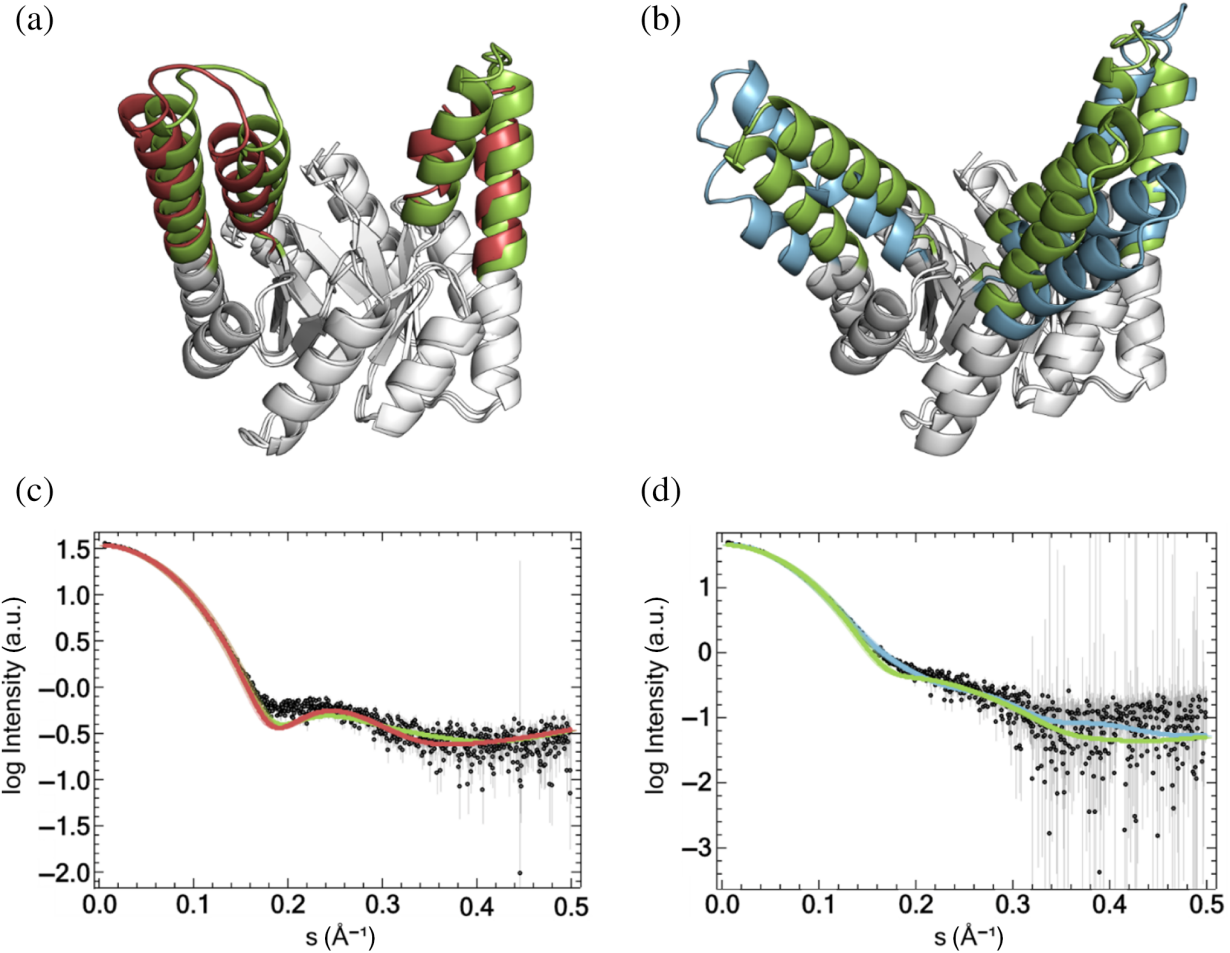
Structural comparison and size exclusion chromatography small angle x‐ray scattering (SEC‐SAXS) analysis of the crystal structures and structure predictions. All structures are displayed as cartoon representation and superimposed over all Cα atoms. The base scaffold is shown in white. Extensions of the structure predictions are colored green. Extensions of HalluTIM2‐2 and HalluTIM3‐1 are shown in red and blue, respectively. SEC‐SAXS experimental scattering data are displayed as black dots. Theoretical scattering curves for the structures are shown in the same color code as in the structural comparison. (a) Superimposition of the experimentally determined and the predicted structures of HalluTIM2‐2 (RMSD: 1.2 Å). (b) Superimposition for HalluTIM3‐1 (RMSD: 4.1 Å). (c) SEC‐SAXS data analysis and comparison to the structures for HalluTIM2‐2 (crystal structure *χ* ^2^: 2.6, AlphaFold2 prediction *χ* ^2^: 2.4). (d) SEC‐SAXS data analysis for HalluTIM3‐1 (crystal structure *χ* ^2^: 2.0, AlphaFold2 prediction *χ* ^2^: 8.9).

Upon comparison of the obtained crystal structures to corresponding structure predictions using ColabFold, we observed an accurate prediction for HalluTIM2‐2 with a root mean square deviation (RMSD) over all Cα atoms of about 1.2 Å but found major differences in the case of HalluTIM3‐1 as the RMSD over all Cα atoms is over 4.1 Å (Figure 4a,b). These discrepancies are mainly due to the different angles of the extensions from the barrel core, especially for insertion 1 that does not form a continuous α‐helix. The structure prediction shows straighter extensions, whereas two of the extensions in the crystal structure tilt more to the outside. When comparing each individual extension with the corresponding prediction, we observe accurate predictions below 1.0 Å RMSD except for the first extension of HalluTIM3‐1, which shows a higher deviation with 2.34 Å (Table S4). To obtain an impression of the protein structure in solution, we measured size exclusion chromatography small angle x‐ray scattering (SEC‐SAXS) with both constructs and sTIM11‐SB. The experimental data indicate globular proteins, whereby both HalluTIMs show a slightly higher flexibility in comparison to the base scaffold (Figure S4). For a comparison with the structures, we calculated a theoretical scatter curve for each crystal structure as well as each prediction and fitted it to the experimental curve (Franke et al., 2017). In the case of HalluTIM2‐2, the theoretical scatter curves of both the crystal structure and the predicted structure are in overall agreement with the experimental data, with a *χ* ^2^ of 2.6 and 2.4, respectively (Figure 4c). However, around 0.18 Å^−1^ both theoretical scatter curves diverge from the experimental data, suggesting a potential high flexibility in the extensions, which is especially conceivable for the partially resolved one. For HalluTIM3‐1, where the crystal structure and prediction differ, we obtained varying qualities of the fits. The theoretical scattering curve of the predicted structure shows a high *χ* ^2^ of 8.9, whereas the crystal structure matches the experimental data with a significantly lower *χ* ^2^ of 2.0 (Figure 4d), indicating that the crystal structure matches the protein in solution more closely.

Next, we searched for newly introduced pockets within the crystal structures employing the AI‐based ligand‐binding site prediction tool PUResNET (Kandel et al., 2021). When analyzing the starting scaffold, a shallow pocket is predicted near the N‐ and C‐termini above the inner β‐sheet (Figure S5A). In contrast, in HalluTIM2‐2 and HalluTIM3‐1, a major pocket is formed through the introduced fragments above the C‐terminal end of the inner β‐sheet (Figure S5B,C). These pockets show differences in size with pocket volumes of 1006 Å^3^ in HalluTIM3‐1 and 2000 Å^3^ in HalluTIM2‐2 (Table S5).

## 3 DISCUSSION

Despite the rapidly growing number of *de novo* TIM barrels, the designs lack the feature of extended surfaces or cavities necessary to introduce catalytic function. We used the recently developed AI‐based method of constrained hallucination from Wang et al. to introduce new structural features on top of the TIM barrel topology (Wang et al., 2022). The insertion sites were selected based on the already successful introduction of different secondary structure elements into a descendant of sTIM11 (Kordes et al., 2023; Wiese et al., 2021), whereby not all three insertion sites had been used simultaneously so far. Through the introduction of two or three extensions, we aimed to generate extended surfaces that allow the formation of cavities. The methods of inpainting and constrained hallucination can both be used to generate insertions with comparable quality. We chose constrained hallucination over inpainting as it is stated to lead to increased structural variability (Wang et al., 2022). In our setup, rather than attaining significant structural diversity, we instead observed only the elongation of the outer α‐helix plus a second smaller α‐helix forming a hairpin located above the barrel. The bias toward helical extensions might be due to the already‐existing helix serving as a seed. To generate greater diversity, the newly developed RFdiffusion application might now be utilized to explore more variable insertion sites across all βα‐loops, thereby encompassing a broader range of insertion lengths (Watson et al., 2023). As RFdiffusion was only published after we completed our computational workflow, which generated high‐quality designs, we did not consider restarting the design process with RFdiffusion. Since helix–loop–helix motifs can build up a cavity, as demonstrated before (Kordes et al., 2023), we continued with our designs and optimized the sequence of the inserted fragment with proteinMPNN. Sequence optimization was focused on the extensions rather than the entire barrel to preserve the structurally robust scaffold, thereby providing a set of diversified HalluTIM variants.

## 4 CONCLUSION

Constrained hallucination in combination with proteinMPNN is a powerful method for the extension of protein loops. Here, we introduced two or three helical insertions into minimal loops in the *de novo* designed TIM barrel sTIM11‐SB. Six HalluTIMs were selected for experimental testing. All of them were found in the soluble fraction after expression in *E. coli*, possibly promoted by the preservation of the base scaffold. Moreover, all HalluTIMs showed a monomeric state and an increased hydrodynamic radius compared to sTIM11‐SB. Multiple HalluTIMs revealed an increase in α‐helicity by CD spectroscopy, indicating the formation of α‐helical extensions. Upon analysis of protein stability, we observed that the extensions in some cases even led to stabilization, indicating the robustness of HalluTIMs for further downstream functionalization. As we were able to introduce three extensions, we attempted to introduce an additional fourth extension to build up the cavity further. Following the symmetry of the already successfully introduced extensions, the fourth one would be located at the termini of the TIM barrel. However, any attempt to build a similar extension by elongation of the termini with constrained hallucination was not successful. The introduced extensions did not show any interactions and rather extended separately away from the rest of the protein (Figure S6). This suggests that elongation of the termini is a more challenging design task for constrained hallucination than the other used insertion sites.

Two of the designs could be crystallized and their structures determined, which we consider an incredible success rate. The crystal structures validate the successful incorporation of the hallucinated extensions. A high amount of crystal contacts could be observed within the introduced α‐helices. This can be rationalized by the sequence optimization with proteinMPNN, which is suggested to generate protein surfaces more likely to form crystal contacts (Wicky et al., 2022). SAXS measurements support the crystal structure despite variations to the structure predictions. Some variation between crystal and solution structure can, however, be expected due to the inherent flexibility of the elongated helical hairpins.

In another study, we used a highly rational and physics‐based approach (Kordes et al., 2023) to incorporate helix–loop–helix motifs into a similar scaffold. Despite entirely different workflows, the resulting designs share similar extensions, pocket formation (Table S5), and the same distinct relationship to natural TIM barrels within a DALI database search, for example, class II fructose‐bisphosphate aldolase (Holm, 2022). Differences can be found in the success rate of the two design workflows. The design workflow by Kordes et al. (2023) generated four designs, of which only two showed soluble expression and no structure could be solved. In contrast, our machine learning‐based design workflow exclusively produced soluble proteins and two structures could be solved providing structural data necessary for future design of ligand‐binding or enzymatic sites.

The TIM barrel sTIM11 was already used for functionalization by fusing one half of the barrel to a *de novo* designed ferredoxin, which dimerizes and binds a lanthanide (Caldwell et al., 2020). In contrast to this functionalized protein, we preserved the TIM‐barrel fold in a monomeric fashion, thereby providing a continuous scaffold to explore a broader spectrum of functions based on the potential of natural TIM barrels (Nagano et al., 2002).

## 5 MATERIALS AND METHODS

### 5.1 Biochemical materials

All reagents were analytical grade from Sigma‐Aldrich or Carl Roth, except when indicated. All solutions were prepared with double‐distilled water. Constructs were codon optimized by BioCat and ordered already cloned in pET21b(+) vector.

### 5.2 Computational extension of a *de novo* TIM barrel

For the modeling and analysis of the extensions into sTIM11‐SB (PDB‐ID: 7OSU), the constrained hallucination method from Wang et al. (2022) was used. During all design steps, the backbone position and amino acid identity of the residues not involved in the design process were restricted. For an initial round of constrained hallucination different combinations of βα‐loops of sTIM11‐SB were chosen as insertion sites. For each insertion site, extensions in the range of 25–35 residues were allowed. One‐hundred were modeled using 600 steps of gradient descent. The resulting designs were relaxed and scored using Rosetta (Leaver‐Fay et al., 2011). Structures of the designs were predicted with AlphaFold2 using the Model 4 weights (Jumper et al., 2021). Designs were filtered based on their average predicted local distance difference test (pLDDT) and Rosetta scores. The best design was passed on for a second round of constrained hallucination. Hereby, the insertion site was chosen between the top of the outer hallucinated α‐helix and the end of the β‐strand of the TIM barrel. The range of an allowed extension was shortened to 19–26 residues. Modeling and filtering were performed identical to the first round of constrained hallucination. Based on a visual inspection of the top scoring designs, particularly with respect to the transition region from the outer α‐helix of the barrel to the extension and the packing of the α‐helix extensions against each other, designs were chosen for a sequence optimization with proteinMPNN (Dauparas et al., 2022). For each chosen backbone, 16 sequences with the full protein backbone model and a temperature factor of 0.2 were generated, whereby everything except the extensions were restricted to their original amino acid identities. For all generated sequences, structures were predicted using ColabFold (v1.3.0) with all five model weights (Mirdita et al., 2022). The prediction with the highest average pLDDT score was selected as the final structure prediction for this sequence. Based on these pLDDT scores and visual inspection as described above, designs were chosen for experimental characterization (Tables S1 and S2).

### 5.3 Overexpression and protein purification

*E. coli* BL21(DE3) cells (Novagen) were transformed with plasmid, plated on agar plates containing 100 μg mL^−1^ ampicillin, and incubated over night at 37°C. From these plates, single colonies were picked to inoculate Lysogeny Broth (LB) media supplemented with ampicillin (100 μg mL^−1^) and incubated at 30°C overnight. For protein expression, 1 L LB was inoculated with 10 mL of the preculture and incubated at 37°C until OD_600_ reached a value of 0.6–0.8. Overexpression was induced by adding isopropyl‐β‐thiogalactoside to a final concentration of 0.1 mM. Cultures were further incubated at 20°C overnight. On the next day, cells were harvested by centrifugation (Beckman Coulter Avanti J‐26 XPI, JLA‐8.1000, 15 min, 4000 g, 4°C) and pellets were either frozen at −20°C until usage or directly resuspended in 35 mL of buffer A (35 mM of NaP pH 8.0, 150 mM of NaCl, and 10 mM of imidazole). The resuspended cells were lysed by sonication (Branson Ultrasonic Sonifier 250, output 4, duty cycle 40%, 3 × 3 min) and centrifuged (Beckman Coulter Avanti J‐26 XPI, JA‐25.50, 1 h, 40,000 g, 4°C). The supernatant was loaded onto a HisTrapHP column (5 mL, Cytiva Life Science) equilibrated with buffer A and coupled to an ÄKTApure system (Cytiva Life Science). After washing with 10 column volumes (CV) of buffer A, the protein was eluted with a linear gradient over 20 CV to 60% buffer B (35 mM of NaP pH 8.0, 150 mM of NaCl, and 500 mM imidazole). Fractions containing the protein were pooled, concentrated with a centrifugal concentrator, and loaded onto a HiLoad 26/600 Superdex 75 preparative grade column (Cytiva Life Sciences) preequilibrated in buffer C (35 mM of NaP pH 8.0, 150 mM of NaCl). Elution was performed with 1 CV buffer C. Fractions with monomeric protein were pooled. For some subsequent experiments, the protein was dialyzed into buffer D (10 mM of NaP, pH 8). Protein concentration was determined photometrically using the absorption at 280 nm. Expression and purification were checked by sodium dodecyl sulfate‐polyacrylamide gel electrophoresis (SDS‐PAGE).

### 5.4 Size exclusion chromatography‐multi angle light scattering

SEC‐MALS measurements were performed using a Superdex 75 Increase 10/300 GL column (Cytiva Life Sciences) connected to an Agilent 1260 Infinity II HPLC system, coupled to a miniDAWN MALS detector and an Optilab differential refractive index detector (dRI) (Wyatt Technology). For all experiments, a protein concentration of 2 mg mL^−1^, a flowrate of 0.8 mL min^−1^, an injection volume of 100 μL, and buffer C with the addition of 0.02% NaN_3_ were used. Data collection and analysis were performed with the ASTRA 8.0.2.5 software (Wyatt Technology). For the analysis of each run, the signal of the dRI detector was used for protein concentration determination. A bovine serum albumin (BSA) standard at 2 mg mL^−1^ was used for MALS detector normalization, correction of peak alignment, peak broadening, and reproducibility.

### 5.5 Far‐Ultraviolet circular dichroism

CD spectra were collected with a Jasco J‐710. Experiments were performed in buffer D using a protein concentration of 0.2 mg mL^−1^. Far‐Ultraviolet‐CD spectra were recorded in the range of 190–260 nm at 20°C in a 1 mm cuvette, with a 1 nm bandwidth, 1 s response time, and scanning speed of 100 nm min^−1^. For each protein, 10 spectra were accumulated. Data were normalized by subtraction of a buffer spectrum and conversion to mean residue molar ellipticity using: [*θ*_MRE_] = (*M* × *θ*)/(10 × *d* × *c*) and *M* = MW/(*n* − 1), where *M* is the mean residue weight, MW the molecular weight in Da, *n* the number of residues in the protein, *θ* the collected ellipticity in mdeg, *d* the path length in cm, and *c* the protein concentration in mg mL^−1^.

To measure thermostability of the proteins, thermal unfolding was followed by CD at 222 nm. The samples were heated up to 95°C with a rate of 1°C min^−1^. Measured unfolding curves were analyzed with the Denatured Protein function of SpectraAnalysis 1.53.07 (Jasco). Dependencies in the initial and final baselines were fitted and subtracted before unfolding parameters were determined. Each parameter was determined from measurements of two individually purified samples and averaged. Δ*G*_25°C_ values were calculated from the obtained values for Δ*Η* and Δ*S* by using the Gibbs–Helmholtz equation with *T* = 298 K. In addition, spectra were collected after the heating process at 95°C and after cooling to 20°C with the parameters described above.

### 5.6 Crystallization and structure determination

Initial crystallization screens using the sitting drop vapor diffusion method were set up using a Phoenix pipetting robot (Art Robbins Instruments) with commercially available sparse‐matrix screens (NeXtal) in 96‐well sitting‐drop plates (3‐drop Intelli‐Plates, Art Robbins Instruments). Droplets were pipetted in 1:1, 1:2, and 2:1 ratios of protein: reservoir solution with a protein concentration of 25 mg mL^−1^ for HalluTIM3‐1 and 20 mg mL^−1^ for HalluTIM2‐2. Plates were incubated at 293 K. Hits for HalluTIM3‐1 were obtained in 0.08 M sodium acetate pH 4.6, 1.6 M ammonium sulfate, 20% (v/v) glycerol after 3 days and for HalluTIM2‐2 in 0.2 M lithium sulfate, 0.1 M Tris pH 8.6, 25 % polyethylene glycol 8000 after 2 days.

Crystals for HalluTIM2‐2 were further optimized using the initial hit and setting up hanging drops in 15‐well EasyXtal plates (NeXtal). The best diffracting crystals were obtained in the initial condition composition. Cryoprotection was achieved by the addition of glycerol to a final concentration of 25%.

Crystals for HalluTIM3‐1 were further optimized using the initial hit and setting up sitting drops in 48‐well MRC Maxi crystallization plates (Swissci). The best diffracting crystals were obtained in 0.08 M sodium acetate pH 4.9, 1.55 M ammonium sulfate, and 20% (v/v) glycerol.

Crystals were manually mounted using cryo‐loops on SPINE standard bases and flash‐cooled in liquid nitrogen. Diffraction data for HalluTIM3‐1 were collected on P13 operated by European Molecular Biology Laboratory (EMBL) Hamburg at the PETRA III storage ring (DESY, Hamburg, Germany) and for HalluTIM2‐2 on ID30B at the European Synchrotron Radiation Facility (ESRF) electron‐storage ring (Nanao et al., 2022). Measurements were performed at 100 K in single‐wavelength mode at 0.9762 Å with a Dectris EIGER X 16 M for HalluTIM3‐1 and at 0.8731 Å with a Dectris EIGER2 X 9 M detector for HalluTIM2‐2 in fine‐slicing mode in 0.1° and 0.05° wedges, respectively, using the *MXCuBE* beamline‐control software (Oscarsson et al., 2019). Data were processed with X‐ray Detector Software APP3 (*XDSAPP3*) (Sparta et al., 2016) employing *XDS* (Kabsch, 2010). Data quality was assessed by applying *phenix.xtriage* (Liebschner et al., 2019).

Phases were solved by molecular replacement using the respective model as search model with *Phaser* (McCoy et al., 2007). The resulting models were manually rebuilt with *Coot* (Emsley et al., 2010) and refined with *phenix.refine* (Afonine et al., 2012) in an iterative manner. Coordinates and structure factors were validated and deposited in the PDB (Burley et al., 2023) with accession codes 8R8N (HalluTIM2‐2) and 8R8O (HalluTIM3‐1).

### 5.7 Size exclusion chromatography small angle x‐ray scattering

SEC‐SAXS measurements were performed at the BioSAXS beamline BM29 at the ESRF in Grenoble, France. For all experiments, a protein concentration of 5 mg mL^−1^, an AdvanceBio Sec 130 Column with a flowrate of 0.16 ml min^−1^, an injection volume of 50 μL and buffer C with the addition of 1 mM dithiotreitol (DTT) were used. Data processing of the experimental scattering curves and analysis were performed with the software suite ATSAS 3.2.1 and BioXTAS RAW (Hopkins et al., 2017; Manalastas‐Cantos et al., 2021). For each measured protein, a theoretical scattering curve with the crystal structure and the structure prediction was calculated and fitted to the experimental data using CRYSOL with standard parameters (Franke et al., 2017).

## AUTHOR CONTRIBUTIONS

**Julian Beck:** Conceptualization; investigation; methodology; data curation; visualization; writing – original draft; writing – review and editing. **Sooruban Shanmugaratnam:** Investigation; data curation; visualization; writing – original draft; writing – review and editing. **Birte Höcker:** Conceptualization; funding acquisition; writing – original draft; writing – review and editing; resources; methodology.

## FUNDING INFORMATION

This work was supported through core funding of the University of Bayreuth.

## Supporting information


**Data S1.** Supporting Information.
