# Supplementary material for: Diversifying de novo TIM barrels by hallucination
Source: Protein Sci. 2024 May 9;33(6):e5001. doi: 10.1002/pro.5001 (PMC11081422; doi:10.1002/pro.5001)
Supplement: Supplementary file 1 — Data S1. Supporting Information. [file PRO-33-e5001-s001.docx]

# Supporting information

**Diversifying *de novo* TIM barrels by hallucination**

Julian Beck^1^, Sooruban Shanmugaratnam^1^, Birte Höcker^1^*

^1^ Department of Biochemistry, University of Bayreuth, 95447 Bayreuth, Germany.

* Corresponding author:

Birte Höcker, e-mail address: [birte.hoecker@uni-bayreuth.de](mailto:birte.hoecker@uni-bayreuth.de)

This file includes:

- Figure S1. Biochemical characterization of additional HalluTIMs
- Figure S2. Far UV-CD measurements to determine the reversibility of thermal unfolding
- Figure S3. Crystal packing with large void volumes of HalluTIM3-1
- Figure S4. Dimensionless Kratky plot
- Figure S5. PUResNET pocket prediction
- Figure S6. Hallucination of a fourth extension
- Table S1. Protein sequences
- Table S2. Parameters for the final designs
- Table S3. Biochemical and thermodynamic properties
- Table S4. Structural comparisons
- Table S5. Comparison of pocket volumes in extended *de novo* TIM barrels
- Table S6. Data collection and refinement statistics of HalluTIM2-2 and HalluTIM3-1


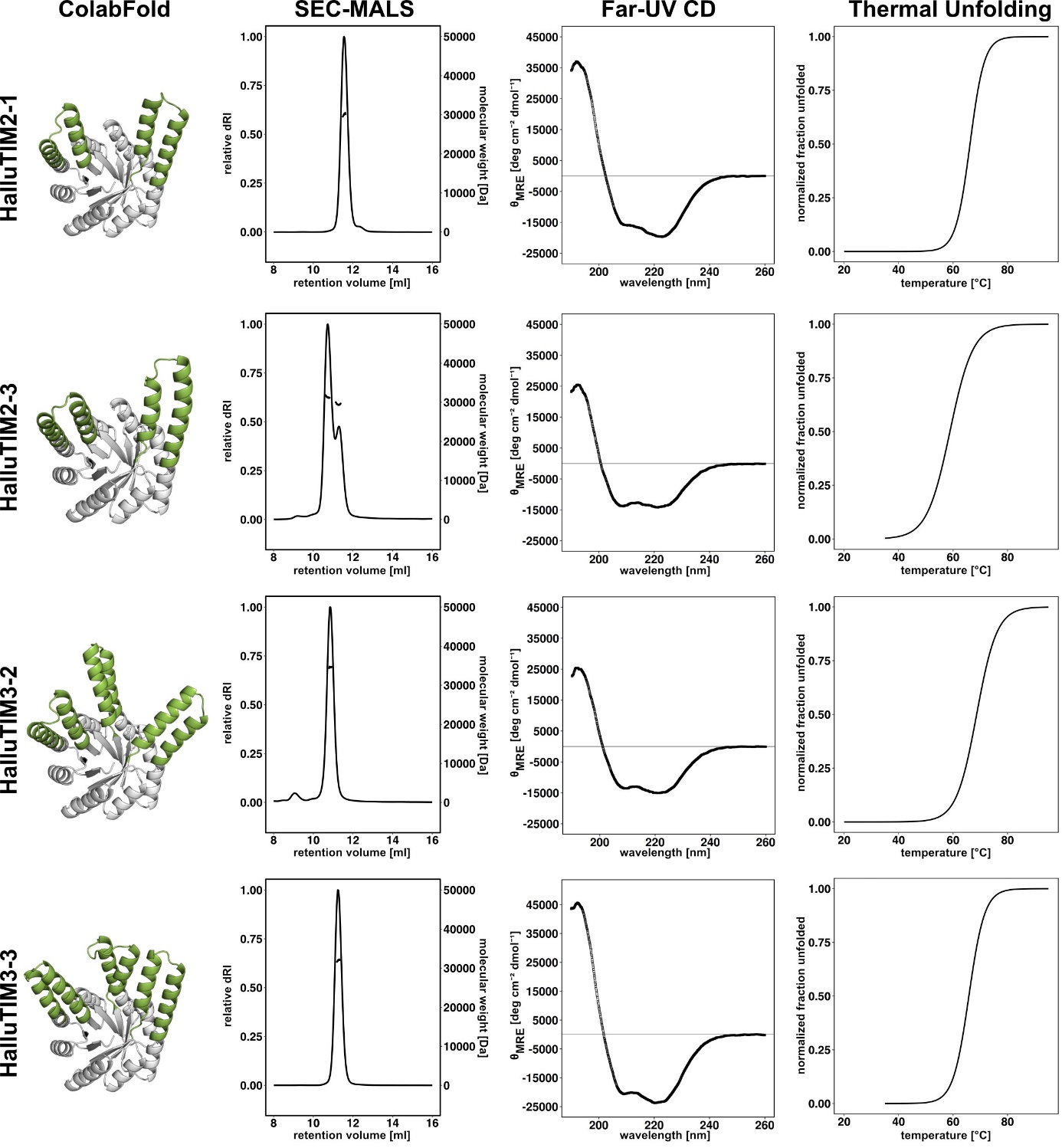


**Figure S1. Biochemical characterization of additional HalluTIMs.** For each construct the structure prediction with ColabFold and the characterization with SEC-MALS, Far UV-CD and thermal unfolding is shown. Within the structure predictions the base scaffold is shown in white and the extensions in green. Elution profile of the SEC-MALS measurements showing the normalized relative differential refractive index as solid black line and the calculated molar mass as data points in black. Far UV-CD spectra and thermal unfolding are displayed in black. For numerical results of the experimental characterization see Table S3.


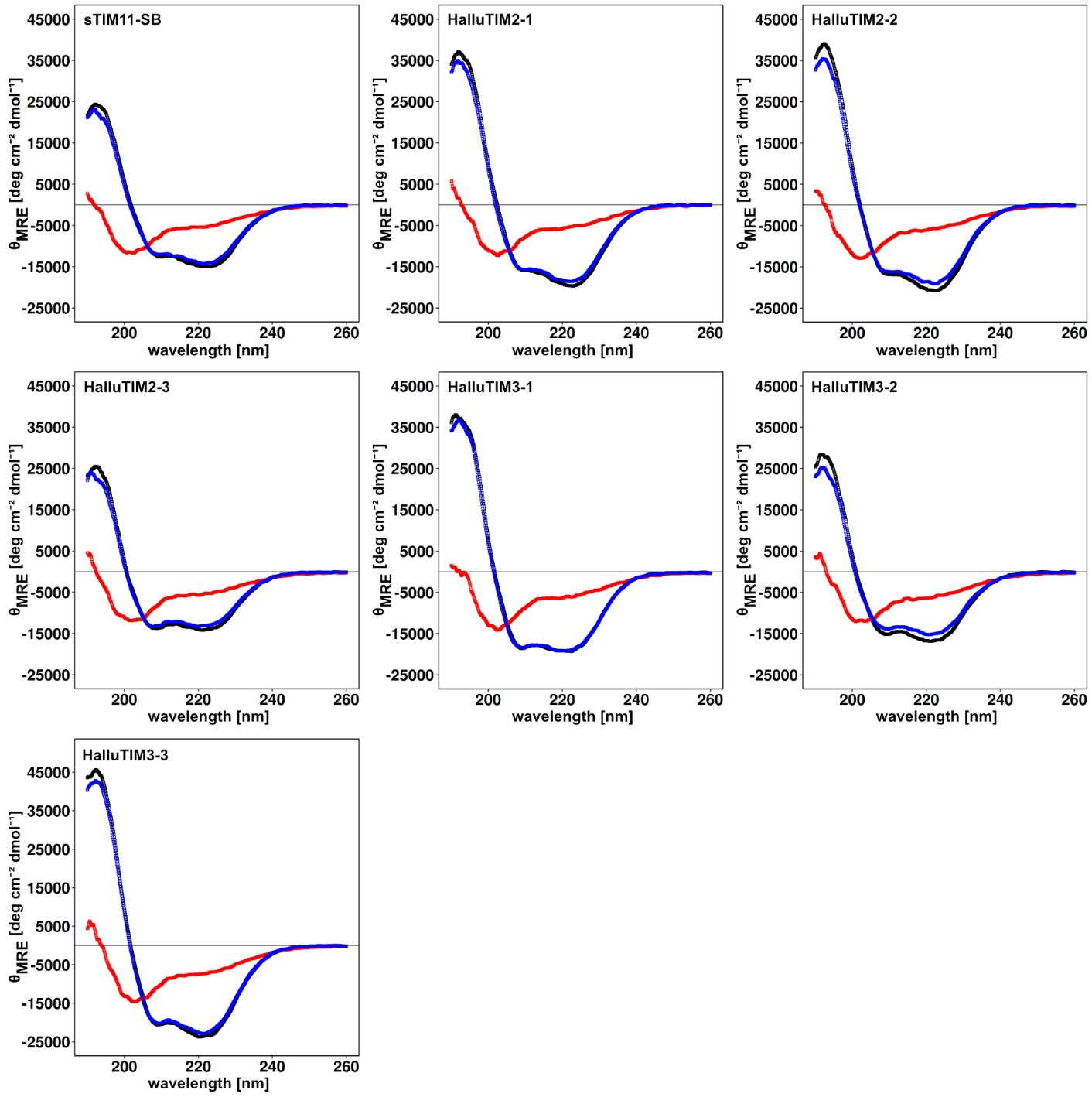


**Figure S2. Far UV-CD measurements to determine the reversibility of thermal unfolding.** For each construct an initial far UV-CD spectrum is displayed in black, a far UV-CD spectrum at 95 °C in red and a far UV-CD spectrum after cooling down in blue. All HalluTIMs maintain the reversible unfolding behavior of sTIM11-SB.


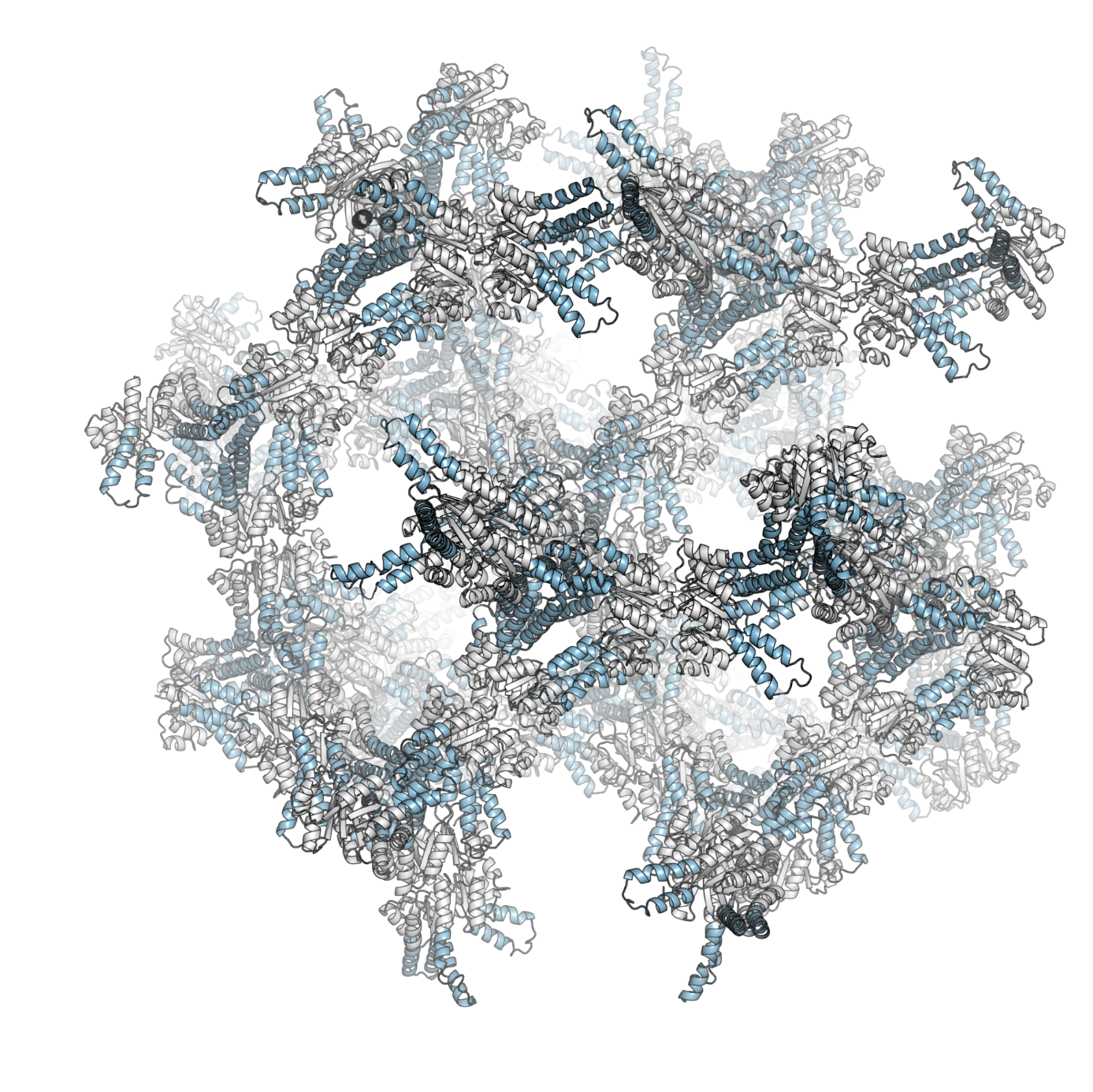


**Figure S3. Crystal packing with large void volumes of HalluTIM3-1.** Structures are displayed as cartoon representation with the base scaffold colored in white and the extensions in blue. Symmetry mates around 100 Å are shown. Crystal contacts are mainly formed within the extensions resulting in a crystal packing with large void volumes.


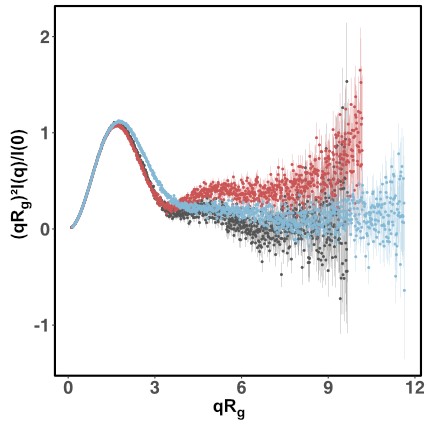


**Figure S4. Dimensionless Kratky plot.** Datapoints and error bars for sTIM11-SB colored in grey, for HalluTIM2-2 in red and for HalluTIM3-1 in blue. Measurements indicate globular proteins and slightly higher flexibility of HalluTIM2-2 and HalluTIM3-1 in comparison to sTIM11‑SB.


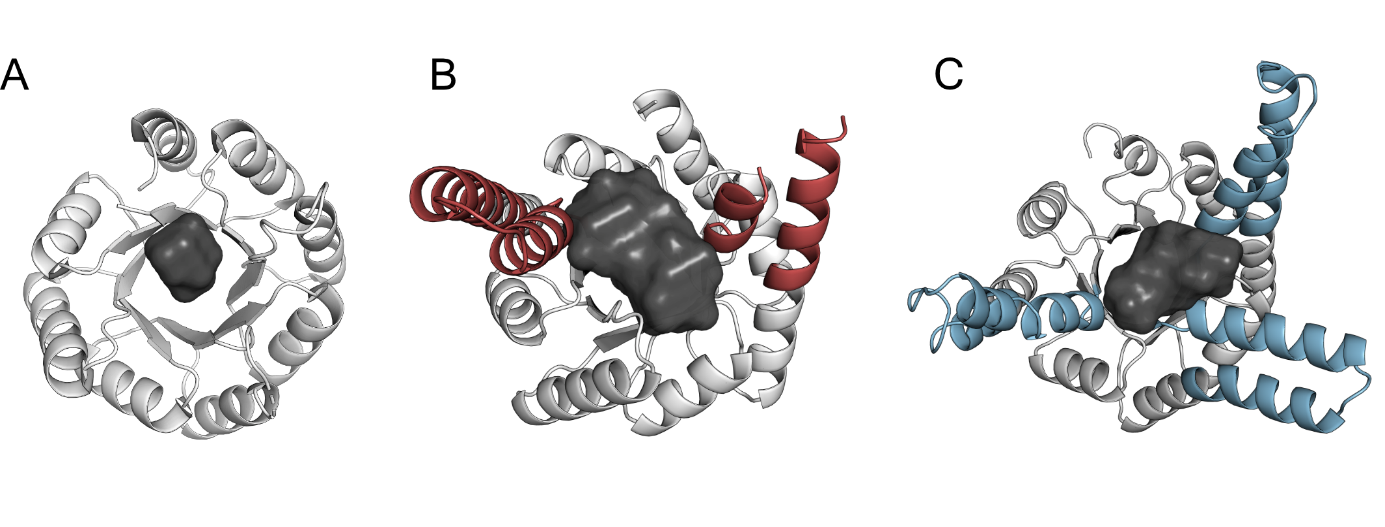


**Figure S5. PUResNET pocket prediction.** Structures are displayed as cartoon representation. The base scaffold is shown in white. Extensions of HalluTIM2‑2 and HalluTIM3‑1 are shown in red and blue, respectively. Predicted pockets are displayed as surface representation and colored in black. **A)** Pocket prediction for sTIM11-SB. **B)** Pocket prediction for HalluTIM2‑2. **C)** Pocket prediction for HalluTIM3-1.


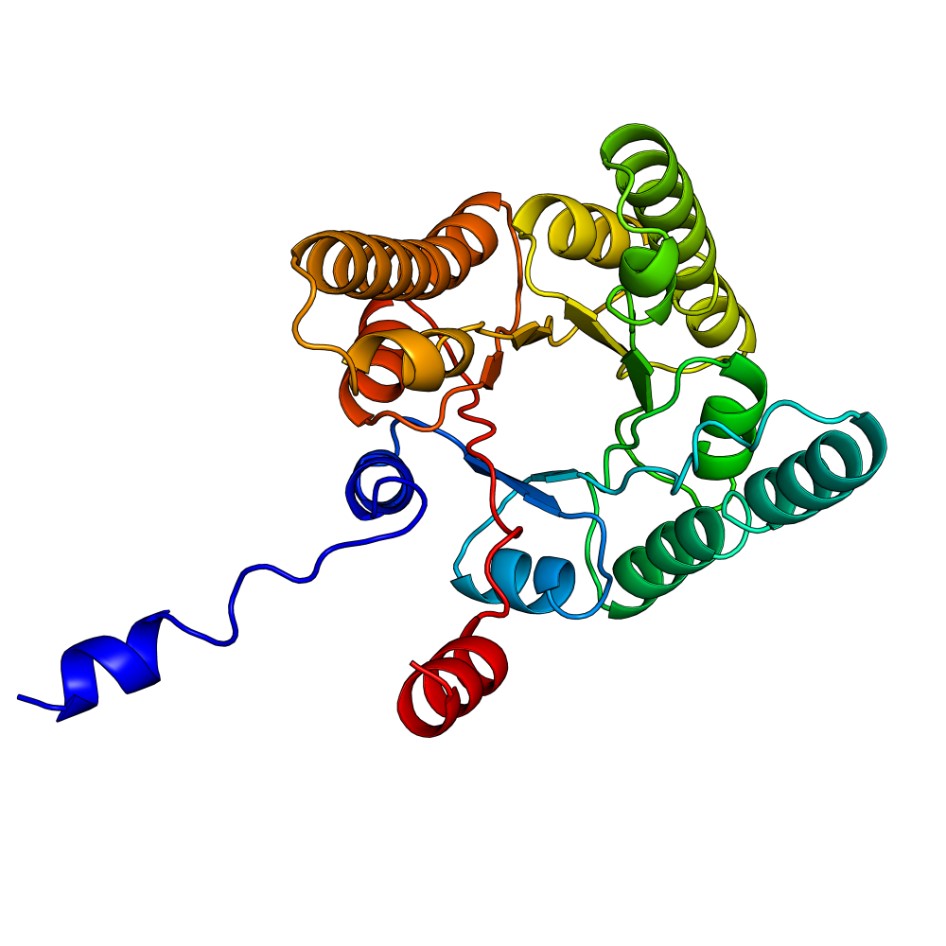


**Figure S6. Hallucination of a fourth extension.** The predicted structure is displayed as cartoon representation in rainbow coloring. The extension of the termini with constrained hallucination resulted only in non-interacting elongations.

**Table S1. Protein sequences** (extensions highlighted in bold).

| Name | Sequence |
| --- | --- |
| HalluTIM2-1 | MDKDEAWKQVEQLRREGATRIAYRSDDWRDLKEAWKKGADILIV**VDKADEYRKKAEEVAKKTGNFKPLVDKYLAEAEKARDEA**WKQVEQLRREGATEIAYRSDDWRDLKEAWKKGADILIVDATDKNEAWKQVEQLRREGATRIAYRSDDWRDLKEAWKKGADILIV**DADERVERRKEELKKLGLTDPEVIEKAREEARREA**WKQVEQLRREGATEIAYRSDDWRDLKEAWKKGADILIVDATLEHHHHHH |
| HalluTIM2-2 | MDKDEAWKQVEQLRREGATRIAYRSDDWRDLKEAWKKGADILIV**SSKADDYRARAAAAAKELGNVKPIVDALLAEAKKARDEA**WKQVEQLRREGATEIAYRSDDWRDLKEAWKKGADILIVDATDKNEAWKQVEQLRREGATRIAYRSDDWRDLKEAWKKGADILIV**DVNARIEKRRKKLAAEGRTDPAVIEAEAAKAREEG**WKQVEQLRREGATEIAYRSDDWRDLKEAWKKGADILIVDATLEHHHHHH |
| HalluTIM2-3 | MDKDEAWKQVEQLRREGATRIAYRSDDWRDLKEAWKKGADILIV**DASRASAALQAAKNAKDPKEKEKLLKENQEKAQKIRDEA**WKQVEQLRREGATEIAYRSDDWRDLKEAWKKGADILIVDATDKNEAWKQVEQLRREGATRIAYRSDDWRDLKEAWKKGADILIV**DADDTADAIRKRAEAEGNKPEYEKKIDEVREKA**WKQVEQLRREGATEIAYRSDDWRDLKEAWKKGADILIVDATLEHHHHHH |
| HalluTIM3-1 | MDKDEAWKQVEQLRREGATRIAYRSDDWRDLKEAWKKGADILIV**DASRLREAADAARAAGEATGDEELIAKAEAYRDEA**WKQVEQLRREGATEIAYRSDDWRDLKEAWKKGADILIV**DGLRRGRIARELERLAKEEGDPALLAAAEAAREAA**WKQVEQLRREGATRIAYRSDDWRDLKEAWKKGADILIV**DNRARLRRAEEEVAETGDPDNEELIRETRERAREEG**WKQVEQLRREGATEIAYRSDDWRDLKEAWKKGADILIVDATLEHHHHHH |
| HalluTIM3-2 | MDKDEAWKQVEQLRREGATRIAYRSDDWRDLKEAWKKGADILIV**DARRKRRAADAAEARGKATGDPEAIAVGQAYRDEA**WKQVEQLRREGATEIAYRSDDWRDLKEAWKKGADILIV**DGVTRRGRARRLRRAAEAEGDPELLAEARALREEA**WKQVEQLRREGATRIAYRSDDWRDLKEAWKKGADILIV**DARTLLRRAREEVAAEGRPDDPELIEKTIAEAREEA**WKQVEQLRREGATEIAYRSDDWRDLKEAWKKGADILIVDATLEHHHHHH |
| HalluTIM3-3 | MDKDEAWKQVEQLRREGATRIAYRSDDWRDLKEAWKKGADILIV**VSKAIEWRAEEAKALAAGDKEAAAKAAAAAKQARDEA**WKQVEQLRREGATEIAYRSDDWRDLKEAWKKGADILIV**ESGEDRTRRRAIELGLFDPNNPEVQKAREEAKQEA**WKQVEQLRREGATRIAYRSDDWRDLKEAWKKGADILIV**DAKSLEEKAEKLLKEAKKRNDPELEKKAEELKKEA**WKQVEQLRREGATEIAYRSDDWRDLKEAWKKGADILIVDATLEHHHHHH |

**Table S2. Parameters for the final designs.** Length in number of residues of each extension in the design (from N- to C-terminus). RMSD over all Cα-atoms for the proteinMPNN input structure and the AlphaFold prediction. pLDDT for each design.

| Design | Extension (# res) | RMSD (Å) | pLDDT |
| --- | --- | --- | --- |
| HalluTIM2-1 | 39/35 | 1.93 | 95.2 |
| HalluTIM2-2 | 39/35 | 1.76 | 95.7 |
| HalluTIM2-3 | 39/33 | 1.74 | 94.4 |
| HalluTIM3-1 | 35/35/36 | 1.93 | 94.6 |
| HalluTIM3-2 | 35/35/36 | 1.93 | 94.9 |
| HalluTIM3-3 | 37/35/35 | 2.25 | 95.0 |

**Table S3.** **Biochemical and thermodynamic properties.** Data points for theoretical and experimentally determined molecular weight (MW) using SEC-MALS as well as apparent melting temperature (T_M_) and ΔG at 25 °C using CD spectroscopy.

| construct | Theoretical MW [kDa] | Experimental MW [kDa] | T_M_ [°C] (n=2) | ΔG_25 °C_ [kcal mol^-1^] |
| --- | --- | --- | --- | --- |
| sTIM11-SB | 22.93 | 22.90 ± 0.05 | 65.5 ± 0.7 | -7.3 ± 0.8 |
| HalluTIM2-1 | 29.77 | 30.10 ± 0.03 | 65.7 ± 0.5 | -10.4 ± 0.2 |
| HalluTIM2-2 | 29.08 | 28.90 ± 0.03 | 71.7 ± 1.1 | -12.3 ± 0.1 |
| HalluTIM2-3 | 29.23 | 31.30 ± 0.09 / 29.50 ± 0.18 | 58.3 ± 1.0 | -5.1 ± 0.1 |
| HalluTIM3-1 | 31.96 | 33.00 ± 0.03 | 66.5 ± 0.7 | -10.5 ± 0.4 |
| HalluTIM3-2 | 32.00 | 34.60 ± 0.21 | 68.4 ± 0.3 | -7.2 ± 0.8 |
| HalluTIM3-3 | 32.16 | 32.00 ± 0.06 | 65.1 ± 0.7 | -8.9 ± 0.6 |

**Table S4.** **Structural comparisons.** Structural alignment over all Cα-atoms each extension from the crystal structures with the ones in the corresponding ColabFold predictions. Numbering of the helices from N- to C- terminus.

|  | Structural alignment | Number of Cα-atoms |
| --- | --- | --- |
| HalluTIM2-2 | Extension 1: 0.44 Å | 39 of 39 Cα-atoms |
|  | Extension 2: 0.80 Å | 22 of 22 Cα-atoms |
| HalluTIM3-1 | Extension 1: 2.34 Å | 35 of 35 Cα-atoms |
|  | Extension 2: 0.65 Å | 35 of 35 Cα-atoms |
|  | Extension 3: 0.96 Å | 36 of 36 Cα-atoms |

**Table S5. Comparison of pocket volumes in extended *de novo* TIM barrels.** Pocket volumes are calculated with ChimeraX (Meng *et al.*, 2023), the ones for αTIM2 and αTIM2-2 derive from Kordes *et al.* (2023)*.*

| Construct | Pocket volume (Å^3^) |
| --- | --- |
| sTIM11-SB | 316 |
| HalluTIM2-2 | 2000 |
| HalluTIM3-1 | 1006 |
| αTIM2 | 750 |
| αTIM2-2 | 2127 |

Reference: Meng, E. C., Goddard, T. D., Pettersen, E. F., Couch, G. S., Pearson, Z. J., Morris, J. H., & Ferrin, T. E. (2023). UCSF ChimeraX: Tools for structure building and analysis. *Protein Science*, *32*(11), e4792. doi: 10.1002/PRO.4792

**Table S6. Data collection and refinement statistics of HalluTIM2-2 and HalluTIM3-1.** Statistics for the highest-resolution shell are shown in parentheses.

|  | **HalluTIM2-2** | **HalluTIM3-1** |
| --- | --- | --- |
| Wavelength | 0.8731 | 0.9762 |
| Resolution range | 47.63 - 2.55 (2.641 - 2.55) | 38.23 - 2.15 (2.227 - 2.15) |
| Space group | P 21 2 21 | P 64 2 2 |
| Unit cell | 92.71 71.2 88.64 90 90 90 | 122.36 122.36 165.62 90 90 120 |
| Total reflections | 80245 (8237) | 1586693 (164305) |
| Unique reflections | 19247 (1921) | 40404 (3948) |
| Multiplicity | 4.2 (4.3) | 39.3 (41.6) |
| Completeness (%) | 97.11 (96.36) | 99.54 (95.95) |
| Mean I/sigma(I) | 8.98 (0.70) | 17.65 (0.46) |
| Wilson B-factor | 91.96 | 67.89 |
| R-merge | 0.07381 (1.654) | 0.1358 (5.774) |
| R-meas | 0.08514 (1.89) | 0.1376 (5.845) |
| R-pim | 0.04118 (0.8896) | 0.02208 (0.8997) |
| CC1/2 | 0.997 (0.302) | 0.999 (0.342) |
| CC* | 0.999 (0.681) | 1 (0.714) |
| Reflections used in refinement | 19246 (1879) | 40401 (3790) |
| Reflections used for R-free | 958 (92) | 2008 (186) |
| R-work | 0.2465 (0.3853) | 0.2453 (0.4822) |
| R-free | 0.3060 (0.4170) | 0.2598 (0.4945) |
| CC(work) | 0.960 (0.474) | 0.939 (0.587) |
| CC(free) | 0.866 (0.050) | 0.936 (0.504) |
| Number of non-hydrogen atoms | 3800 | 2289 |
| macromolecules | 3777 | 2192 |
| ligands | 15 | 47 |
| solvent | 8 | 50 |
| Protein residues | 461 | 262 |
| RMS(bonds) | 0.003 | 0.002 |
| RMS(angles) | 0.5 | 0.44 |
| Ramachandran favored (%) | 97.57 | 97.69 |
| Ramachandran allowed (%) | 1.77 | 1.92 |
| Ramachandran outliers (%) | 0.66 | 0.38 |
| Rotamer outliers (%) | 5.45 | 1.9 |
| Clashscore | 7.27 | 6.76 |
| Average B-factor | 110.94 | 91.96 |
| macromolecules | 110.96 | 91.72 |
| ligands | 112.21 | 106.54 |
| solvent | 102.33 | 89.13 |
| Number of TLS groups | 2 | 3 |
